# Global Control of Phosphotransferase System-Mediated Carbon Metabolism by CRP Is Associated with Metabolic Homeostasis and Virulence in *Klebsiella pneumoniae*

**DOI:** 10.3390/microorganisms14040882

**Published:** 2026-04-14

**Authors:** Shumin Liu, Yiting Guan, Yan Zhang, Min Niu, Kai Yang, Yan Du

**Affiliations:** 1 Department of Clinical Laboratory, The First Affiliated Hospital of Kunming Medical University, Kunming 650032, China; shuminhappy@126.com (S.L.); gyt18213364672@163.com (Y.G.); 18908853416@163.com (Y.Z.); 15198724701@139.com (M.N.); kaiyang201@126.com (K.Y.); 2 Yunnan Province Clinical Research Center for Laboratory Medicine, Kunming 650032, China; 3 Yunnan Key Laboratory of Laboratory Medicine, Kunming 650032, China

**Keywords:** phosphotransferase system, cyclic AMP receptor protein, virulence, homeostasis, *Klebsiella pneumoniae*

## Abstract

The cyclic AMP receptor protein (CRP) is a highly conserved global transcriptional regulator that integrates carbon metabolism and environmental adaptation in bacteria. However, its systematic role in the regulation of virulence in *K. pneumoniae* remains poorly understood; In this study, we constructed a *crp* deletion mutant (Δ*crp*) and a complemented strain (c-Δ*crp*) and employed a combination of in vitro virulence assays, in vivo infection models, transcriptomic profiling, and functional metabolic analyses to dissect the CRP-mediated metabolism–virulence regulatory axis; We show that *crp* deficiency does not significantly alter susceptibility to clinically relevant antibiotics but markedly impairs biofilm formation, motility, and host cell adhesion and invasion. In murine infection models, the Δ*crp* strain exhibits significantly reduced pulmonary colonization and lethality. Transcriptomic analysis reveals broad downregulation of phosphotransferase system (PTS)-associated genes, including *srlA*/*srlB*/*srlE*, *mtlA* and *malX*. Functional assays further demonstrate that *crp* loss severely compromises growth on multiple host-relevant carbon sources and is accompanied by aberrant accumulation of intracellular ATP and NADH, indicative of disrupted metabolic homeostasis; Collectively, these findings identify crp as an important regulator associated with PTS-mediated carbon metabolic balance, and virulence-related phenotypes in *K. pneumoniae*. Accordingly, targeting the CRP–PTS axis may offer a theoretical basis for metabolism-oriented anti-virulence interventions against *K. pneumoniae* by attenuating pathogenicity.

## 1. Introduction

*K. pneumoniae* is a major Gram-negative opportunistic pathogen responsible for abroad spectrum of severe infections, including pneumonia, bacteremia, urinary tract infections, and liver abscesses, particularly in immunocompromised and hospitalized patients [1,2]. Over the past decade, the rapid global dissemination of carbapenem-resistant *K. pneumoniae* (CRKP) has posed a critical threat to public health and has been designated by the World Health Organization as a priority pathogen for urgent research and drug development [3,4]. CRKP remains highly prevalent worldwide, with significant regional variations. A multicentre molecular epidemiological study in China revealed that CRKP persisted and spread between 2011 and 2021, with the ST11 clade emerging as the predominant circulating strain. Specifically, the ST11-KL64 serotype is predominant in CRKP, with a detection rate as high as 59.5% in China. Bacterial genome mutations, such as in antimicrobial resistance, virulence, and metabolism-associated genes, may be associated with the high prevalence of this subclone [5,6]. Concurrently, the ST15-KL47 and ST307 clonal lineages have disseminated extensively. Publicly available genomic analyses have confirmed the global spread of the ST11-KL64 type of CRKP [7]. For instance, the genomic sequence analysis of 794 CRKP isolates collected from bloodstream infections at 40 Chinese hospitals between 2014 and 2019 revealed subclonal replacement within the dominant ST11 clone [8]. Among pediatric patients, data from a children’s hospital in Shanghai for the period 2017–2021 show that the predominant sequence type for CRKP infections shifted from ST278-NDM-1 to ST11-KPC-2 [9]. Studies on CRKP in paediatric patients in Henan, China, have also revealed its unique epidemiological characteristics [10]. In addition to its extensive antibiotic resistance, CRKP frequently exhibits high pathogenic potential, relying on efficient colonization, dissemination, and adaptation to the complex and dynamic host microenvironment [11]. However, the upstream regulatory mechanisms that drive CRKP virulence, particularly the mechanistic links between metabolic regulation and virulence expression, remain poorly understood.

Increasing evidence indicates that bacterial virulence is tightly coupled to metabolic state and nutrient sensing rather than being regulated as an independent module [12,13]. During infection, pathogens encounter highly heterogeneous [14] and often nutrient-limited niches within host tissues, necessitating precise control of carbon acquisition and metabolic flux to sustain energy production and biosynthetic demands while activating virulence-associated functions such as motility [15], biofilm formation [16], and host cell interaction [17]. In this context, metabolic regulation has emerged as a central integrative node linking environmental adaptation to pathogenic potential.

The PTS represents one of the most important carbohydrate transport and regulatory systems in Gram-negative bacteria [18,19]. Beyond mediating the uptake and phosphorylation of diverse sugars and sugar alcohols, the PTS network is functionally integrated with global regulatory circuits that coordinate metabolic flux distribution and signal transduction [20]. Recent studies suggest that PTS activity is intimately associated with multiple virulence-related processes, including capsule biosynthesis, biofilm formation, and host immune evasion [21]. Nevertheless, the functional contribution of the PTS system to virulence regulation in *K. pneumoniae*, particularly in CRKP, and its upstream transcriptional control remain largely unexplored. The cyclic AMP receptor protein (CRP) is a highly conserved, cAMP-dependent global transcriptional regulator that serves as a key contributor in bacterial carbon metabolism [22,23,24]. By sensing intracellular cAMP levels, CRP orchestrates the expression of genes involved in carbon utilization, energy metabolism, and stress responses, thereby enabling adaptive responses to fluctuating environmental conditions [25,26]. Notably, accumulating evidence across diverse bacterial pathogens has revealed that CRP not only influences metabolic programs but also directly or indirectly modulates virulence-associated phenotypes, positioning it as a critical molecular link between metabolic state and pathogenic behavior.

In *Escherichia coli* and *Salmonella* spp., CRP has been shown to regulate bacterial motility, secretion systems, and host cell invasion, thereby shaping infection efficiency and disease progression [27,28,29]. In *Vibrio cholerae,* CRP is associated with carbon metabolism with virulence gene expression to control cholera toxin production and intestinal colonization [30,31]. Similarly, in *Pseudomonas aeruginosa*, CRP homologs have been implicated in the regulation of pathogenicity islands, quorum sensing, biofilm formation, and multiple virulence determinants [32,33]. Collectively, these studies underscore a conserved role for CRP in the metabolic control of virulence, while also highlighting species-specific regulatory architectures and environmental dependencies.

In contrast, systematic investigations into the role of CRP in *K. pneumoniae* remain limited and have primarily focused on its function in carbon metabolism and growth regulation [34,35]. Whether CRP-driven metabolic reprogramming contributes to virulence-associated phenotypes [36,37], and whether CRP acts as a regulatory nexus linking carbon source utilization [38], metabolic homeostasis [39], and in vivo pathogenicity in CRKP, remain open questions.

Here, this study identifies CRP as an important regulator that orchestrates the growth, metabolic rewiring, and pathogenesis of *K. pneumoniae*. By characterizing *crp* deletion and complementation strains through a combination of transcriptomic profiling, carbon utilization assays, and in vivo infection models, we demonstrate that CRP maintains cellular energy homeostasis by modulating the PTS-centered carbon uptake network. This regulatory axis contributes to coordinating metabolic adaptability with the expression of virulence factors. Our findings provide a molecular framework for understanding the high pathogenicity of CRKP from the perspective of metabolic integration, offering a theoretical foundation for developing anti-virulence strategies targeting bacterial metabolism.

## 2. Materials and Methods

### 2.1. Bacterial Strains and Culture Conditions

The strains used in this study were CRKP-27 (WT), its isogenic *crp* gene deletion mutant (Δ*crp*), and the complementation strain (c-Δ*crp*). All strains were routinely cultured at 37 °C in Luria–Bertani (LB) liquid medium or LB agar plates. Antibiotics were added as required based on plasmid selection markers. For carbon utilization assays, bacteria were grown in M9 minimal medium supplemented with 0.2% (*w*/*v*) of either glucose, maltose, sorbitol, or mannitol.

### 2.2. Cell Culture

The TC-1 cell line was routinely cultured in Dulbecco’s Modified Eagle Medium (DMEM) (Corning Inc., Corning, NY, USA) supplemented with 10% fetal bovine serum (FBS) (Corning Inc., Corning, NY, USA) and penicillin- streptomycin (Thermo Fisher Scientific Inc., Waltham, MA, USA) at 100 U/mL each. The cell cultures were incubated at 37 °C in a humidified 5% carbon dioxide incubator.

### 2.3. Animal Model

The C57BL/6J mice (male, 8 weeks) were obtained from Charles River Laboratories Inc., Wilmington, MA, USA. All mice were housed in a specific pathogen-free (SPF) facility at 24 °C temperature and 45–55% humidity on a regular 12 h light/dark cycle and were provided with autoclaved food and water at all times.

### 2.4. Construction of the Δcrp Mutant and Complemented Strains

The *crp* gene was deleted using the CRISPR-Cas9 genome editing system. Using CRKP-27 genomic DNA as a template, the upstream and downstream flanking homology arms were amplified with primers *crp*-Up-F/R and *crp*-Down-F/R, respectively, and subsequently fused via overlap extension PCR. The resulting repair template was verified by sequencing. To initiate editing, the pTcCas9 plasmid was electroporated into the WT strain and selected on LB agar containing 10 μg/mL tetracycline. Competent cells harboring pTcCas9 were then co-transformed with the repair template and the sgRNA expression vector. Transformants were screened on LB agar supplemented with tetracycline (10 μg/mL) and gentamicin (10 μg/mL). The crp mutants were identified via PCR using primers *crp*-Up-F/Down-R and *crp*-ter-F/R, followed by Sanger sequencing of the junction fragments. For genetic complementation, the crp fragment and the pCMTac plasmid backbone were amplified using primers *crp*-ptac-F/R and ptac-*crp*-F/R, respectively. The purified amplicons were assembled via seamless cloning to generate the complementation vector, which was then electroporated into the Δ*crp* strain. The c-Δ*crp* strain was selected on LB agar with 34 μg/mL chloramphenicol and validated by PCR (primers pCMtac-F/M13R) and sequencing. The primers used were listed Table S1.

### 2.5. Antimicrobial Susceptibility Assays

Minimum inhibitory concentrations (MIC) of meropenem (GlpBio Technology Inc., Montclair, CA, USA), imipenem (GlpBio Technology Inc., Montclair, CA, USA), and ceftriaxone (GlpBio Technology Inc., Montclair, CA, USA) were determined using the broth microdilution method, in accordance with the Clinical and Laboratory Standards Institute (CLSI) guidelines. Bacteria (5 × 10^5^ CFU/mL, 100 μL) were inoculated into 96-well plates containing various concentrations of antibiotics. After 18–20 h of incubation at 37 °C, the MIC was defined as the lowest concentration that achieved complete inhibition of visible growth [40].

### 2.6. Biofilm Formation Assays

Biofilm formation was quantified using the crystal violet staining method [41]. Briefly, bacterial cultures were inoculated into 96-well polystyrene plates and incubated statically at 37 °C for 24 h. Following incubation, the supernatant was removed, and the wells were washed with 0.2 M phosphate-buffered saline (PBS, PH = 7.4). The biomass was stained with 0.1% (*w*/*v*) crystal violet for 15 min, after which the bound dye was solubilized in 95% ethanol. Biofilm formation was then quantified by measuring the absorbance at OD570.

### 2.7. Swimming Motility Assays

Swimming motility assay was performed as previously described [42]. A bacterial suspension adjusted to an OD600 of 1.0 was centrally inoculated onto semi-solid LB agar plates (0.3% *w*/*v* agar). Following spot inoculation, the plates were incubated at 37 °C for 24 h. The diameter of the halo formed in the agar was then measured using the ImageJ software (v1.53t).

### 2.8. Adhesion and Invasion Assays

TC-1 cells were maintained in DMEM supplemented with 10% FBS. Upon reaching approximately 80% confluency, the cell monolayers were challenged with bacteria at a multiplicity of infection (MOI) of 100 for 1 h. After incubation, the cells were washed with 0.2 M PBS (PH = 7.4). to remove non-adherent bacteria. For invasion quantification, the extracellular bacteria were eliminated by treating the monolayers with 100 μg/mL gentamicin for an additional 1 h. Finally, the host cells were lysed, and the intracellular bacteria were quantified by plating serial dilutions of the lysates onto LB agar for colony counts enumeration.

### 2.9. Electron Microscopy (EM) Analysis

Bacterial samples were fixed overnight with 2.5% glutaraldehyde at 4 °C, followed by secondary fixation in 1% osmium tetroxide. Following a graded ethanol dehydration series, samples were processed according to the microscopy modality: specimens for scanning electron microscopy (SEM) underwent critical point drying, while those for transmission electron microscopy (TEM) were embedded in epoxy resin and processed into ultrathin sections. Specimens were sputter-coated and imaged using SEM or TEM [40].

### 2.10. Mice Infection Model

C57BL/6J mice (6–8 weeks old) were randomly assigned to groups. For the intranasal infection model, mice were anaesthetized and inoculated intranasally with 1 × 10^8^ CFU/50 μL bacteria. For the intravenous infection model, 1 × 10^7^ CFU/100 μL bacteria was administered via tail vein injection. The survival rates and clinical signs of illness were monitored daily. At designated time points, the mice were euthanized to collect blood and organs (lung, liver and spleen). These samples were processed for quantifying bacterial burden and histopathological analysis.

### 2.11. Transcriptomic Analysis

Total RNA was extracted from WT and Δcrp strains during the mid-logarithmic growth phase using the TRIzol reagent (Thermo Fisher Scientific Inc., Waltham, MA, USA). RNA integrity and quality were assessed using an Agilent Bioanalyzer (Agilent Technologies Inc., Santa Clara, CA, USA). Following library preparation, paired-end sequencing was performed on the Illumina platform. Differential gene expression analysis was conducted using DESeq2 software (v1.42.0) with the following selection criteria: |log_2_ Fold Change| ≥ 1 and false discovery rate (FDR) < 0.05. Pathway enrichment analysis was conducted using the Kyoto Encyclopedia of Genes and Genomes (KEGG) database to elucidate the metabolic networks regulated by CRP.

### 2.12. Quantitative Real-Time PCR (qRT-PCR)

Total RNA was reverse-transcribed into cDNA using a commercial reverse transcription kit (Applied Biological Materials Inc. Richmond, BC, Canada). Quantitative PCR was performed using a SYBR Green-based detection system, with the 16S rRNA gene serving as the internal reference for normalization. Relative gene expression levels were calculated by BlasTaq™ 2× qPCR MasterMix kit (Applied Biological Materials Inc., Richmond, BC, Canada) on the LightCycler96 Real-Time PCR System (Roche Diagnostics GmbH, Basel, Switzerland, LightCycler96). The primers used were listed in Supplemental Table S1.

### 2.13. Intracellular ATP and NADH Analysis

The bacteria were collected during the mid-logarithmic growth phase. Intracellular ATP (Solarbio Life Sciences, Beijing, China) and NADH (Beyotime Biotechnology, Shanghai, China) levels were determined using commercial bioluminescent and colorimetric assay kits, respectively. Following bacterial lysis, the assays were conducted according to the manufacturers’ protocols. All metabolite concentrations were normalized to the total protein content of the samples to ensure comparability across strains.

### 2.14. Hematoxylin and Eosin (H&E) Staining Assays

Mouse lung samples were collected and fixed in 4% paraformaldehyde at room temperature for 24 to 48 h. Following paraffin embedding, samples were prepared and stained with H&E.

### 2.15. Enzyme-Linked Immunosorbent Assays (ELISA)

The levels of tumor necrosis factor-α (TNF-α) (Dogesce Biotechnology, Shanghai, China), interleukin-1β (IL-1β) (Dogesce, DG30045M-96T), and interleukin-6 (IL-6) (Dogesce, DG30062M-96T) in mouse plasma were quantified using ELISA kits (USCN Life Science Inc., Wuhan, Hubei, China) according to the manufacturers’ instructions.

### 2.16. Carbon Utilization Assays

Overnight bacterial cultures were washed twice with sterile 0.2 M PBS (PH = 7.4) and then inoculated into fresh media supplemented with various carbon sources to monitor growth kinetics.

### 2.17. Statistical Analysis

All experiments were performed in at least three independent biological replicates. Data are presented as the mean ± standard deviation (SD). Unpaired *t*-tests were used when only two groups were compared. Multiple sample comparison was analyzed by one-way analysis of variance (ANOVA), with post hoc Dunnett’s tests used to obtain *p*-values. Differences were considered significant at *p* < 0.05.

## 3. Results

### 3.1. CRP Regulates Growth and Physiological Phenotypes of K. pneumoniae

**Figure 1:**
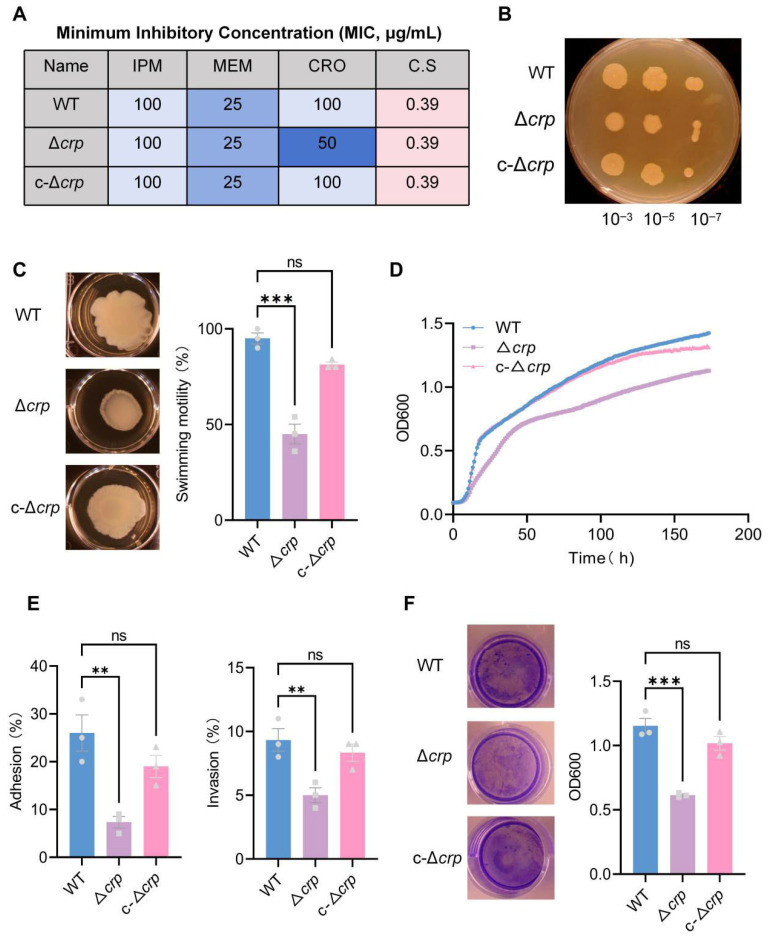
CRP regulates physiological and virulence-associated phenotypes in *K. pneumoniae*. (**A**) Minimum inhibitory concentrations (MICs) of meropenem (MEM), imipenem (IPM), ceftriaxone (CRO), and C.S for the wild-type strain (WT), *crp* deletion mutant (Δ*crp)*, and complemented strain (c-Δ*crp*). (**B**) Representative colony morphologies of the indicated strains grown on solid medium. (**C**) Growth curves of WT, Δ*crp* and c-Δ*crp* strains cultured in LB medium, monitored by OD600 over time. (**D**) Quantification of bacterial adhesion to and invasion into lung epithelial cells. (**E**) Swimming motility of the indicated strains assessed on 0.3% semi-solid agar and corresponding quantitative analysis. (**F**) Biofilm formation measured by crystal violet staining and quantified as OD600. Data are presented as mean ± SD from at three independent experiments. Statistical significance was determined by one-way ANOVA. ns, not significant; ** *p* < 0.01; *** *p* < 0.001.

To determine the role of CRP in basic physiology, we compared the phenotypes of the wild-type (WT), *crp* deletion (Δ*crp*), and complementation (c-Δ*crp*) strains. Antimicrobial susceptibility testing showed no significant differences in MICs for meropenem, imipenem, or ceftriaxone (Figure 1A). However, Δ*crp* formed smaller colonies with a loose structure compared to WT (Figure 1B). Swimming motility assays revealed a significantly reduced expansion radius in the Δ*crp* strain (Figure 1C). Furthermore, Δ*crp* exhibited marked growth retardation in LB broth and reached a lower cell density at the stationary phase. These growth and motility defects were fully restored in the c-Δ*crp* strain, indicating that CRP contributes to normal bacterial fitness (Figure 1D). Electron Microscopy (EM) analysis reveals alterations in morphology and surface architecture in the Δ*crp* strain (Supplementary Figure S1).

### 3.2. CRP Contributes to in Vitro Pathogenicity-Related Phenotypes

We further evaluated the regulatory role of CRP on key pathogenic traits. In a lung epithelial cell model, Δ*crp* showed significantly reduced adhesion and invasion rates compared to the WT (Figure 1E). Additionally, crystal violet staining demonstrated a significant impairment in biofilm formation in the Δ*crp* strain (Figure 1F). The recovery of these phenotypes in the c-Δ*crp* strain suggests that *crp* is a central regulator of host–cell interactions and biofilm development, linking metabolic status to virulence expression.

### 3.3. CRP Deficiency Attenuates in Vivo Virulence and Colonization

**Figure 2:**
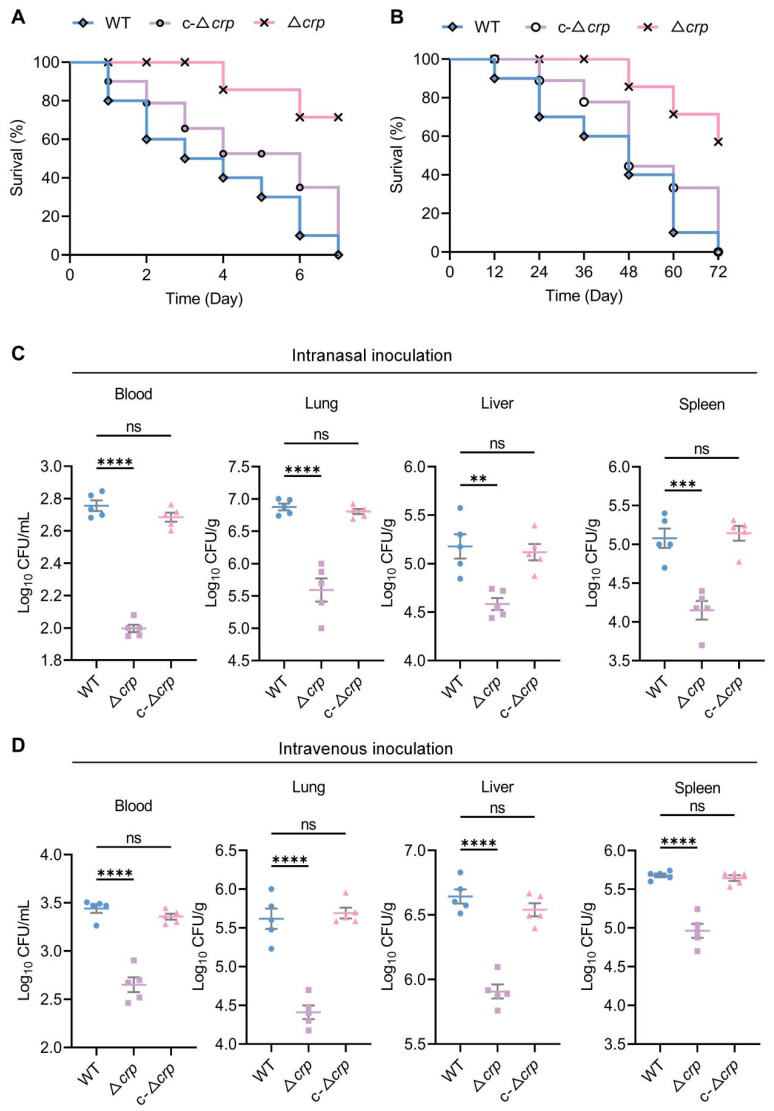
CRP promotes in vivo pathogenicity and host colonization. (**A**,**B**) Survival curves of mice following intranasal (**A**) or intravenous (**B**) inoculation with WT, Δ*crp*, and complemented strains (c-Δ*crp*) (*n* = 10). Mice infected with the Δ*crp* strain exhibited significantly prolonged survival compared with those infected with the WT strain, whereas complementation partially or fully restored virulence. (**C**) Bacterial load in blood, lungs, liver, and spleen of mice 12 h after intranasal infection. (**D**) Bacterial load in blood, lungs, liver, and spleen of mice 12 h after intravenous infection. Data are expressed as mean ± standard deviation (*n* = 5). ns, not significant; ** *p* < 0.01, *** *p* < 0.001, and **** *p* < 0.0001 by unpaired *t*-test.

**Figure 3:**
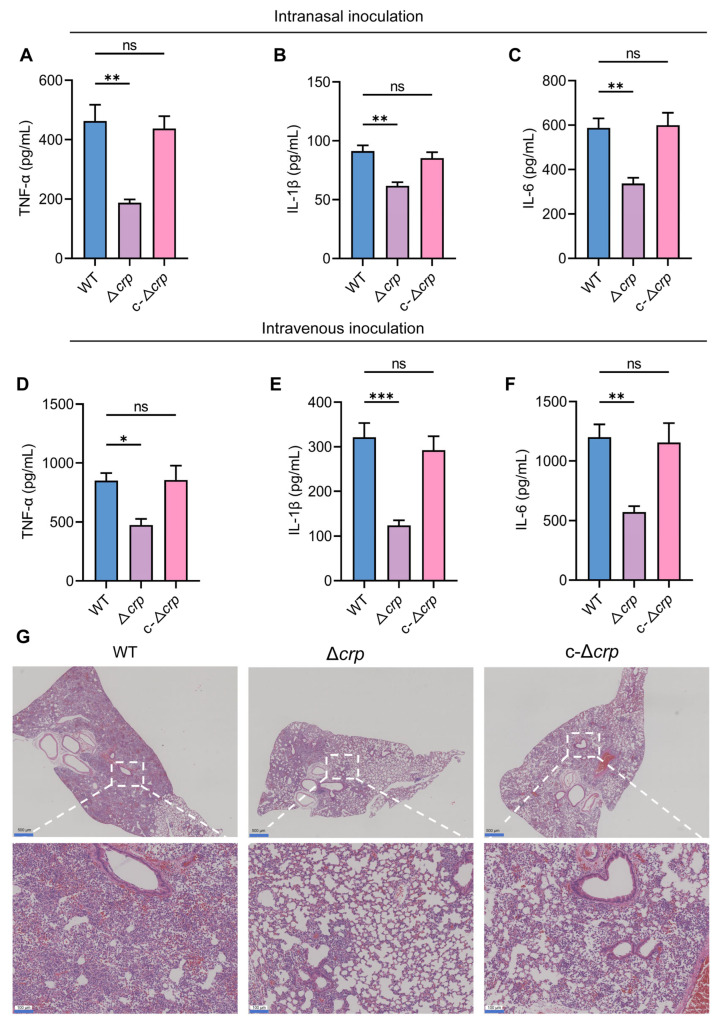
CRP modulates host inflammatory responses and pulmonary pathology during infection. (**A**–**C**) Plasma levels of TNF-α (**A**), IL-1β (**B**), and IL-6 (**C**) in mice following intranasal infection with WT, Δ*crp* and c-Δ*crp* strains. (**D**–**F**) Plasma levels of TNF-α (**D**), IL-1β (**E**), and IL-6 (**F**) following intravenous infection. (**G**) Representative hematoxylin and eosin (H&E)-stained lung sections showing extensive inflammatory cell infiltration and alveolar structural disruption in WT-infected mice, markedly attenuated pathology in Δ*crp*-infected mice and restoration of tissue injury in c-Δ*crp*-infected mice. Data are expressed as mean ± standard deviation (*n* = 5), * *p* < 0.05, ** *p* < 0.01, and *** *p* < 0.001 by unpaired *t*-test. Statistical significance is indicated as ns (not significant).

The role of CRP in systemic infection was assessed using intranasal and intravenous murine models. Survival analysis showed that Δ*crp* infection significantly increased survival rates of mice (Figure 2A,B). Organ bacterial load analysis (CFU) confirmed that the Δ*crp* strain was severely impaired in primary lung colonization and subsequent systemic dissemination to the blood, liver, and spleen (Figure 2C,D). Consistent with the reduction in bacterial colonization, mice infected with the Δ*crp* strain exhibited significantly decreased plasma levels of inflammatory cytokines, whereas no significant difference was observed between the c-Δ*crp* strain infection group and the wild-type control group (Figure 3A–E). Histopathological examination (H&E staining) revealed extensive inflammatory infiltration and alveolar destruction in WT-infected mice, whereas Δ*crp*-infected mice showed minimal lung injury (Figure 3G). These data identify CRP as a core regulator of *K*. *pneumoniae* lethal virulence.

### 3.4. CRP Reshapes Metabolic Networks via the PTS System

**Figure 4:**
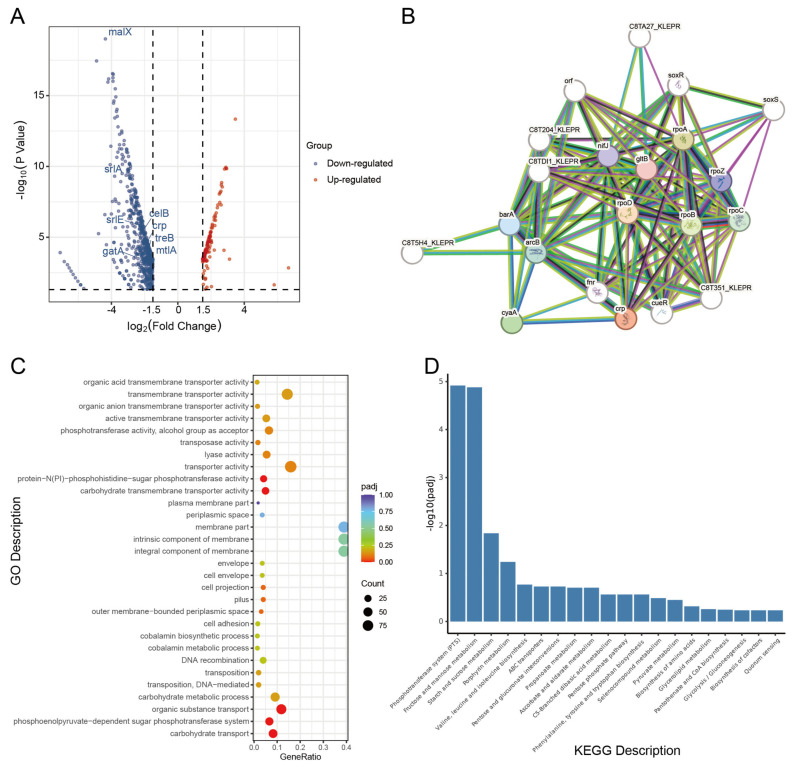
*CRP* knockout drives metabolic reprogramming in *K*. *pneumoniae*, modulating PTS pathways through broad metabolic effects. (**A**) Volcano plot of differentially expressed genes (DEGs) between the *CRP* knockout and wild-type strains. Blue and red dots indicate down- and up-regulated genes, respectively; representative PTS-related genes are labeled. (**B**) Protein–protein interaction (PPI) network of CRP and major differentially expressed regulatory proteins, showing CRP-associated transcriptional and transcriptional regulatory modules. (**C**) Gene Ontology (GO) enrichment analysis of DEGs, highlighting terms related to carbohydrate and organic acid transport, phosphotransferase activity, membrane components, and cobalamin biosynthesis. (**D**) KEGG pathway enrichment analysis of DEGs, with predominant enrichment in the PTS, carbohydrate metabolism, porphyrin metabolism, and amino acid biosynthesis.

**Figure 5:**
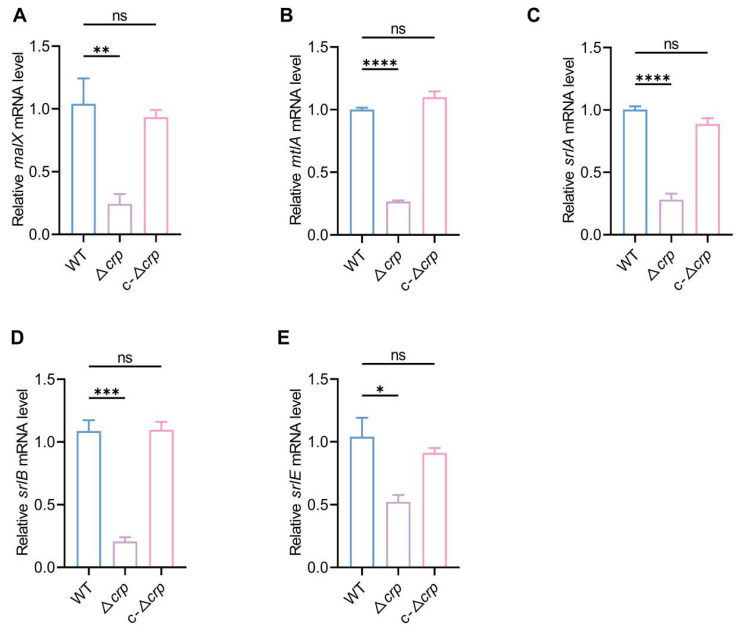
Transcriptomic profiling reveals CRP-dependent repression of the PTS carbon metabolic network. (**A**–**E**) Quantitative real-time PCR (qRT-PCR) validation of representative PTS-related genes, including *srlA*, *srlB*, *srlE*, *malX*, and *mtlA*, confirming significant transcriptional downregulation in the Δ*crp* strain relative to WT and restoration in the complemented strain (c-Δ*crp*). Expression levels were normalized to 16S rRNA and calculated using the 2^−ΔΔCt^ method. Data represent mean ± SD of three independent experiments. * *p* < 0.05, ** *p* < 0.01, *** *p* < 0.001, and **** *p* < 0.0001 by unpaired *t*-test. Statistical significance is indicated as ns (not significant).

Differential expression analysis revealed extensive transcriptional changes following *crp* deletion in *K*. *pneumoniae* (Figure 4A). In addition to the expected loss of *crp* expression, multiple genes involved in carbohydrate transport and utilization were among the most significantly altered genes, including the PTS-associated gene *malX* and several sugar transport-related genes such as *srlA*, *srlE*, *srlB*, *celB*, *treB*, *mtlA*, and *gatA*. Protein–protein interaction analysis further identified genes functionally associated with CRP, with major connected nodes including RNA polymerase subunits (*rpoA*, *rpoB*, *rpoC*, and *rpoD*), as well as *gltB* and the two-component system regulator *barA* (Figure 4B). Gene Ontology enrichment analysis of these CRP-associated genes showed significant enrichment in transcription-related biological processes, including transcription, DNA-templated, regulation of transcription, DNA-templated, nucleobase-containing compound biosynthetic process, and regulation of cellular process (Supplementary Figure S2). Consistently, GO enrichment of all differentially expressed genes indicated that *crp* deletion significantly affected functions related to phosphotransferase activity, carbohydrate and organic acid transport, and membrane-associated components (Figure 4C). KEGG pathway enrichment analysis further demonstrated that differentially expressed genes were predominantly enriched in PTS, multiple carbohydrate metabolism pathways, porphyrin metabolism, and amino acid biosynthesis (Figure 4D). To experimentally validate the transcriptomic findings, quantitative real-time PCR (qRT-PCR) was performed on representative PTS-related genes, including *srlA*, *srlB*, *srlE*, *malX* and *mtlA.* Consistent with RNA-seq data, all five genes exhibited significant transcriptional downregulation in the Δ*crp* strain relative to the WT, while their expression levels were largely restored in the complemented strain (c-Δ*crp)* (Figure 5A–E). This concordance between transcriptomic and qRT-PCR results confirms the robustness of the observed repression of PTS-mediated carbon transport pathways upon *crp* deletion.

### 3.5. CRP Deficiency Impairs Host-Related Carbon Utilization and Disrupts Metabolic Homeostasis

**Figure 6:**
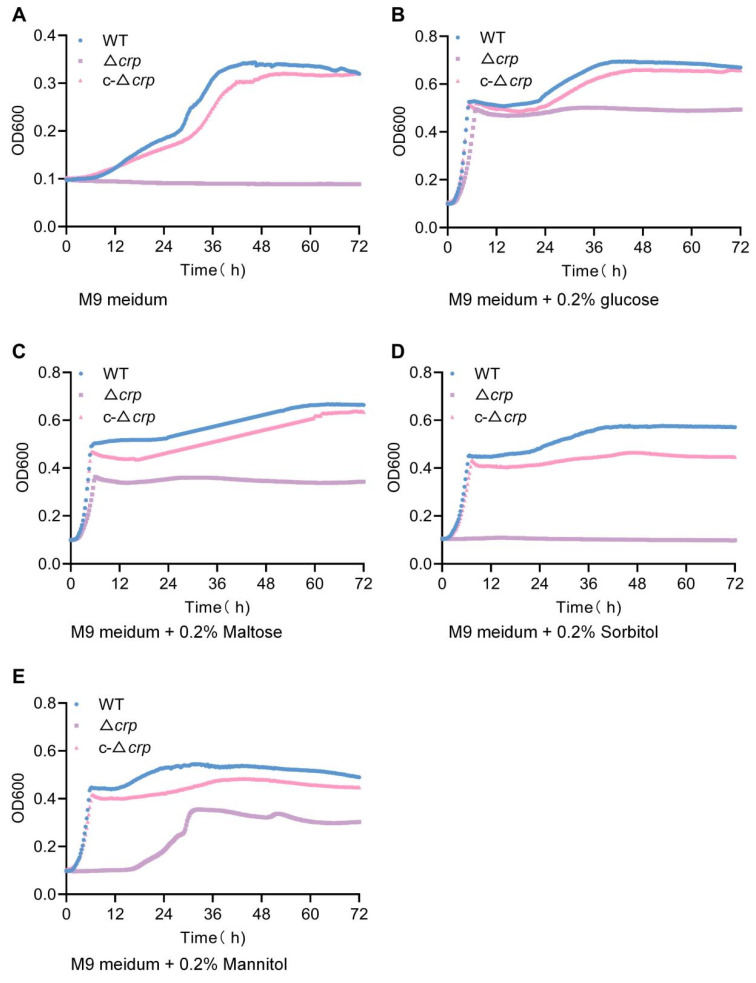
CRP influences carbon source-dependent growth adaptation. (**A**–**E**) Growth curves of WT, Δ*crp* and c-Δ*crp* strains in M9 minimal medium supplemented with different carbon sources, including glucose, maltose, sorbitol, and mannitol. The Δ*crp* strain exhibited growth comparable to WT in glucose- and maltose-supplemented media but showed severely impaired growth when sorbitol or mannitol was provided as the sole carbon source.

To assess the functional consequences of PTS pathway repression, bacterial growth kinetics were monitored in M9 minimal medium supplemented with defined carbon sources. In the presence of glucose or maltose, the Δ*crp* strain retained limited growth capacity, although with a markedly reduced growth rate compared to the WT and complemented strains (Figure 6A,B). In contrast, when sorbitol or mannitol—carbon sources whose uptake and phosphorylation depend on PTS components encoded by srl and mtl operons—were provided as the sole carbon source, the Δ*crp* mutant exhibited an almost complete loss of proliferative ability. Restoration of CRP expression in the complemented strain fully rescued this growth defect (Figure 6C,D). These phenotypes are in strong agreement with the transcriptional repression of *srlA*, *srlB*, *srlE* and *mtlA*, directly linking CRP-dependent regulation of PTS transporter genes to the bacterium’s capacity to exploit alternative, host-associated carbon sources. This functional coupling underscores the role of CRP as a key determinant of metabolic flexibility and ecological fitness under nutrient-limited conditions.

Furthermore, quantitative analysis of intracellular metabolic indicators at uniform cell densities revealed that the Δ*crp* strain harbored significantly higher levels of ATP and NADH compared to the WT and complemented strains (Supplementary Figure S3).

This phenomenon suggests that the absence of CRP leads to a profound disruption of metabolic homeostasis, characterized by the paradoxical accumulation of energy molecules and reducing equivalents. This imbalance indicates a disruption in the intracellular metabolic balance within the Δ*crp* mutant.

## 4. Discussion

In this study, we establish CRP as a central regulatory hub that links carbon metabolism to virulence expression in *K. pneumoniae* [43,44]. Our transcriptomic and functional analyses demonstrate that *CRP* deficiency leads to coordinated repression of a broad PTS-mediated carbon acquisition network [45], thereby limiting the pathogen’s ability to exploit diverse nutrient sources within the host environment. This regulatory pattern aligns with the classical catabolic inhibition paradigm, wherein CRP-cAMP activates alternative carbon utilization pathways in the absence of preferred substrates, yet extends it to reveal a key dimension of *K. pneumoniae*’s virulence effects [46].

Functionally, the inability of the Δ*crp* strain to grow on sorbitol and mannitol underscores the importance of CRP-controlled PTS modules in metabolic adaptation to host-associated niches. The concomitant accumulation of intracellular ATP and NADH suggests a state of metabolic imbalance in which energy production and consumption become uncoupled from biosynthetic and virulence-associated processes [6]. This contradictory energy surplus may be indicative of impeded carbon flux in downstream catabolic and anabolic pathways [47,48]. This may consequently result in feedback inhibition and reduced demand for energy-consuming virulence programmes [49].

This metabolic dysregulation is closely associated with pronounced defects in biofilm formation [37], motility [15], host cell interaction, and in vivo colonization, highlighting metabolic homeostasis as a prerequisite for full virulence expression [14]. Our results suggest a potential link between CRP-dependent PTS regulation and virulence, although the direct mechanistic link remains to be further validated. Based on previous reports, we hypothesise that CRP-mediated activation of the PTS pathway maintains energy balance and the supply of precursor molecules, thereby enabling the execution of energy-intensive pathogenic programs such as capsule synthesis [35], type 3 fimbriae expression [50], and effector translocation [48].

Beyond direct activation of PTS-associated genes, our protein–protein interaction analysis suggests that CRP may operate within a broader transcriptional regulatory hierarchy involving RNA polymerase subunits and global regulators such as BarA (Figure 4 and Figure S2). This finding is consistent with previous reports suggesting that CRP may function not merely as a classic regulator of carbon metabolism, but rather as an architectural modulator of transcriptional prioritization under nutrient-limited conditions [51]. Given the enrichment of transcription-related Gene Ontology terms among CRP-associated genes, we speculate that CRP may influence sigma factor competition, promoter accessibility, or transcriptional elongation efficiency, thereby exerting both direct and indirect control over metabolic and virulence gene expression programs. Such multi-layered regulation would allow *K. pneumoniae* to dynamically reallocate transcriptional resources in response to fluctuating carbon availability within host microenvironments.

The observed accumulation of ATP and NADH in the Δ*crp* mutant lends further support to the hypothesis that CRP contributes to coordinating catabolic flux with downstream anabolic and virulence-related demands [42]. In the absence of CRP-mediated carbon uptake optimization, carbon flux may be redirected toward partial or inefficient pathways, resulting in reduced precursor synthesis despite elevated energy pools [51]. This decoupling between redox state and biomass production could impair the synthesis of surface structures, extracellular matrix components, and motility apparatuses, all of which are energetically and metabolically demanding [52]. Therefore, CRP appears to function as a systems-level integrator that synchronizes carbon influx, redox balance, and macromolecular biosynthesis with the execution of pathogenic phenotypes [53].

The present findings indicate that metabolic regulators, such as CRP, may serve as viable targets for the development of antivirulence strategies. Conventional antibiotics are known to inhibit essential growth processes directly [54]; however, interference with CRP-mediated metabolic coordination may attenuate pathogenicity without imposing strong selective pressure for resistance [55,56,57]. The absence of substantial alterations in antibiotic susceptibility in the Δ*crp* strain lends support to the hypothesis that CRP exerts its primary regulatory influence on virulence and metabolic adaptability rather than on intrinsic drug resistance mechanisms. Consequently, the strategic targeting of global metabolic regulators or specific nodes within the CRP–PTS axis may offer a viable approach to neutralising hypervirulent or multidrug-resistant *K. pneumoniae* strains while maintaining microbiota stability.

Collectively, our findings provide a systems-level framework for understanding how global metabolic regulators shape the pathogenic potential of multidrug-resistant *K. pneumoniae* and suggest that targeting metabolic regulatory networks may represent a promising strategy for antivirulence therapy. Future efforts could explore the translational potential of this CRP-centered regulatory axis for countering carbapenem-resistant *K. pneumoniae*, for example by developing small-molecule inhibitors that disrupt CRP-mediated carbon metabolism, thereby attenuating pathogenicity without exerting direct selective pressure for resistance.

## 5. Conclusions

In summary, this study establishes CRP as an important regulator in *K. pneumoniae* that links central carbon metabolism to virulence. Loss of CRP disrupts metabolic homeostasis and PTS gene expression, leading to impaired biofilm formation, motility, and host colonization without affecting antibiotic susceptibility. These findings position CRP as a promising target for antivirulence therapies.

## Data Availability

The original contributions presented in this study are included in the article/Supplementary Material. Further inquiries can be directed to the corresponding author.

## Supplementary Materials

The following supporting information can be downloaded at: https://www.mdpi.com/article/10.3390/microorganisms14040882/s1, Figure S1: Morphological analysis of *CRP*-deficient *K. pneumoniae.* Figure S2: Genes associated with *CRP* in the protein-protein interaction (PPI) network are enriched in transcription and cellular regulation pathways. Figure S3: *CRP* deletion disrupts intracellular energy and redox homeostasis. Table S1: Primers used in this study.
