# Supplementary material for: Global Control of Phosphotransferase System-Mediated Carbon Metabolism by CRP Is Associated with Metabolic Homeostasis and Virulence in Klebsiella pneumoniae
Source: Microorganisms. 2026 Apr 14;14(4):882. doi: 10.3390/microorganisms14040882 (PMC13119289; doi:10.3390/microorganisms14040882)
Supplement: Supplementary file 1 [file microorganisms-14-00882-s001.zip › microorganisms-4217959-supplementary.pdf]

Supplemental Figure

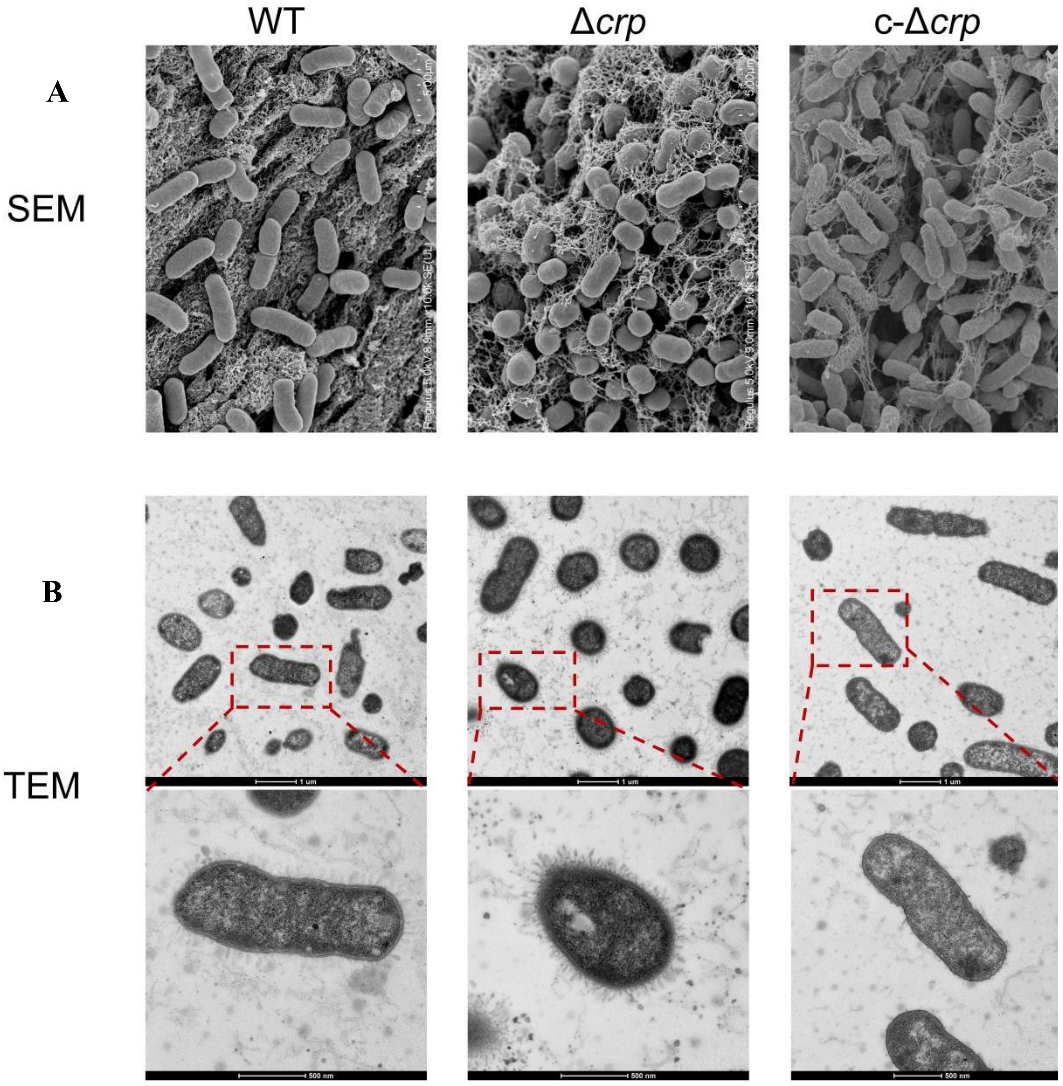

Supplementary Figure S1. Morphological analysis of CRP-deficient *K. pneumoniae***.** (A) Scanning electron microscopy (SEM) images of WT, Δ*crp*, and complemented strains (c-Δ*crp*). (B) Transmission electron microscopy (TEM) images revealing alterations in cell surface architecture in the Δ*crp* strain.

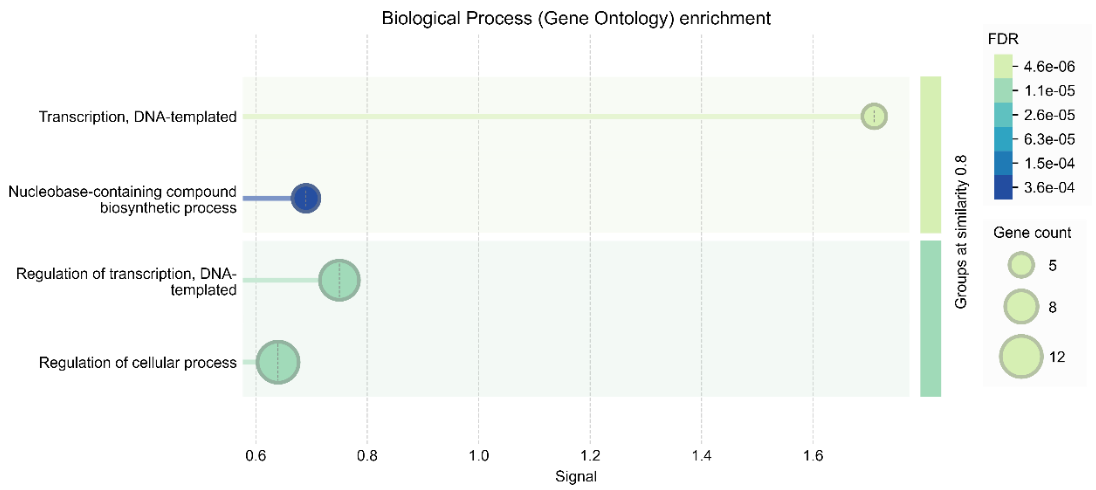

Supplementary Figure S2. Genes associated with CRP in the protein-protein interaction (PPI) network are enriched in transcription and cellular regulation pathways.

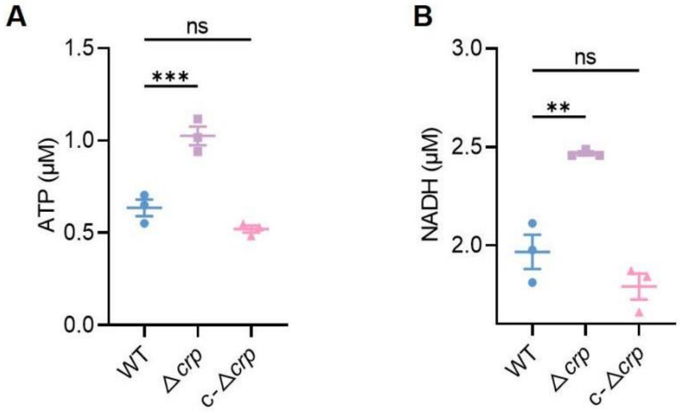

Supplementary Figure S3. CRP deletion disrupts intracellular energy and redox homeostasis. Intracellular ATP and NADH levels in WT, Δ*crp* and c-Δ*crp* strains were measured at mid-logarithmic phase and normalized to total protein content. The Δ*crp* strain exhibited significantly elevated ATP (A) and NADH (B) levels compared with the WT strain, indicating a disruption of metabolic homeostasis and altered utilization of carbon-derived metabolic intermediates. Data represent mean ± SD of three independent experiments. ** $p < 0.01$, and *** $p < 0.001$ by unpaired t-test. Statistical significance is indicated as ns (not significant).

Supplemental Table

Table S1 Primers used in this study.

Primer name	Primer sequence (5′-3′)
crp-Up-F	AGGTCAGCTTCACTTCGCAA
crp-Up-R	caatacgccgcttagcaaGCGCGGTTATCCTCTGTTATA
crp-Down-F	aacagaggataaccgcgcGTTGCTAAGCGGCGTATTGCTCCC
crp-Down-R	TTAAACACGCCGTACCACAG
crp-sgRNA	AAGAGCACGCTGATCCACCA
crp-ter-F	ATGGTGCTTGGCAAACCGCAAAC
crp-ter-R	TTAACGGGTGCCGTAGACGACGA
crp-ptac-F	TACCATGGAAGCTTACATGGTGCTTGGCAAACCGCAAAC A
crp-ptac-R	tgttcgttgaggaattcTTAACGGGTGCCGTAGACGACGAT
ptac-*crp*-F	GTCTACGGCACCCGTTAAgaattcctcaacgaacaaggc
ptac-*crp*-R	GTTTGCCAAGCACCATGTAAGCTTCCATGGTATATCTCCT
pCMtac-F	GAAGCCGGCGGCACCTCGCTAAC
M13R	AGCGGATAACAATTTCACACAGG
entC-RT-F	CCCGGAGCAGGACGCGTTTTTAGC
entC-RT-R	TCGGAGAGCGGCACGTGGAAGTTA
rho-RT-F	AACTACGACAAGCCGGAAAA
rho-RT-R	ACCGTTACCACGCTCCATAC
entC-lacZ-F	ttcacaccggaattcCGGCCAGTCCGCCTGGGCT
entC-lacZ -R	tccgtaatcatggtcatATCATCCTCCAAAAACGATAAG
M13(FAM)-EMSA-F	TGTAAAACGACGGCCAGT
M13-EMSA-R	CCAGTATCGACAAAGGAC
malX-F	GATATTGCCGTCGGTGTTGC
malX-R	TTCCTGTTCCTGTTCGTGGC
mtlA-F	ACCTGAGCCATGTGCGTAAA

mtlA-R	GCAGGTTATTGATGGCGCTG
srlA-F	CTCGGTCGGTTTATGCCAGA
srlA-R	GCGATGCCGAGATAGACGAA
srlB-F	GTGCCTGAAGATGACCGTGA
srlB-R	GCTCGCCGTGGTTGTGAATA
srlE-F	CTACCCCAAGCGTCGTATCC
srlE-R	TGACGCCGGAGACGTAGATA
